# Bis(μ-4-methyl­benzoato)-κ^3^ *O*,*O*′:*O*;κ^3^ *O*:*O*,*O*′-bis­[aqua­(4-methyl­benzoato-κ^2^ *O*,*O*′)(nicotinamide-κ*N* ^1^)cadmium]

**DOI:** 10.1107/S1600536812047046

**Published:** 2012-11-24

**Authors:** Öznur Dincel, Barış Tercan, Efdal Çimen, Hacali Necefoğlu, Tuncer Hökelek

**Affiliations:** a Department of Physics, Karabük University, 78050 Karabük, Turkey; b Department of Chemistry, Kafkas University, 36100 Kars, Turkey; c Department of Physics, Hacettepe University, 06800 Beytepe, Ankara, Turkey

## Abstract

In the dinuclear centrosymmetric title compound, [Cd_2_(C_8_H_7_O_2_)_4_(C_6_H_6_N_2_O)_2_(H_2_O)_2_], the Cd^II^ ion is chelated by two carboxyl­ate groups from 4-methyl­benzoate anions, and is further coordinated by one nicotinamide and one water mol­ecule; a carboxyl­ate O atom from an adjacent 4-methyl­benzoate anion bridges to the Cd^II^ ion, completing the irregular coordination sphere of the seven ligand atoms. In the crystal, inter­molecular O—H⋯O, N—H⋯O and weak C—H⋯O hydrogen bonds link the mol­ecules into a three-dimensional network. The methyl­benzene moiety of one bridging 4-methyl­benzoate anion is disordered over two orientations of equal occupancy.

## Related literature

For niacin, see: Krishnamachari (1974 ▶). For *N*,*N*-diethyl­nicotinamide, see: Bigoli *et al.* (1972 ▶). For related structures, see: Greenaway *et al.* (1984 ▶); Hökelek & Necefoğlu (1996 ▶); Hökelek *et al.* (2009*a* ▶,*b* ▶,*c* ▶,*d* ▶, 2010*a* ▶,*b* ▶); Zaman *et al.* (2012 ▶).
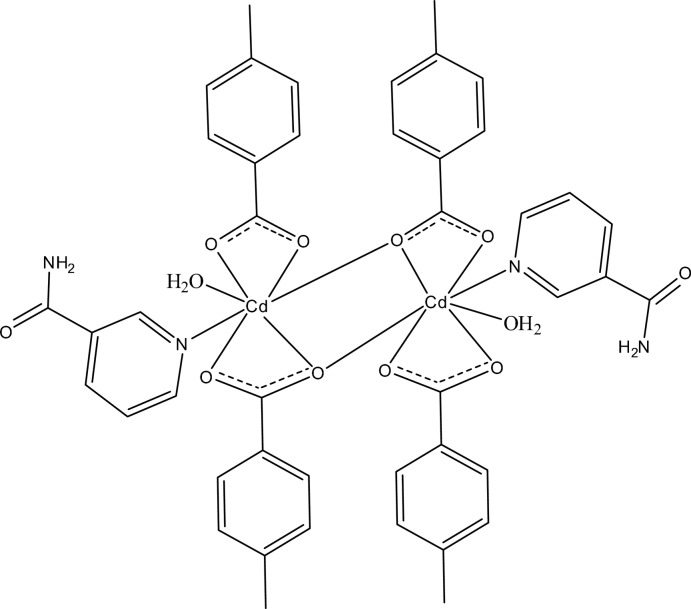


## Experimental

### 

#### Crystal data

[Cd_2_(C_8_H_7_O_2_)_4_(C_6_H_6_N_2_O)_2_(H_2_O)_2_]
*M* *_r_* = 1045.65
Triclinic, 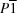

*a* = 9.5935 (2) Å
*b* = 10.3084 (2) Å
*c* = 12.6606 (3) Å
α = 68.326 (3)°
β = 74.999 (3)°
γ = 66.916 (2)°
*V* = 1060.81 (5) Å^3^
*Z* = 1
Mo *K*α radiation
μ = 1.07 mm^−1^
*T* = 100 K
0.36 × 0.31 × 0.28 mm

#### Data collection

Bruker APEXII CCD diffractometer
Absorption correction: multi-scan (*SADABS*; Bruker, 2005 ▶) *T*_min_ = 0.687, *T*_max_ = 0.741
16908 measured reflections
4169 independent reflections
4085 reflections with *I* > 2σ(*I*)
*R*_int_ = 0.021

#### Refinement

*R*[*F* ^2^ > 2σ(*F* ^2^)] = 0.019
*wR*(*F* ^2^) = 0.048
*S* = 1.05
4169 reflections
256 parameters
H-atom parameters constrained
Δρ_max_ = 1.08 e Å^−3^
Δρ_min_ = −0.72 e Å^−3^

### 

Data collection: *APEX2* (Bruker, 2007 ▶); cell refinement: *SAINT* (Bruker, 2007 ▶); data reduction: *SAINT*; program(s) used to solve structure: *SHELXS97* (Sheldrick, 2008 ▶); program(s) used to refine structure: *SHELXL97* (Sheldrick, 2008 ▶); molecular graphics: *Mercury* (Macrae *et al.*, 2006 ▶); software used to prepare material for publication: *WinGX* (Farrugia, 2012 ▶).

## Supplementary Material

Crystal structure: contains datablock(s) I, global. DOI: 10.1107/S1600536812047046/xu5651sup1.cif

Structure factors: contains datablock(s) I. DOI: 10.1107/S1600536812047046/xu5651Isup2.hkl

Additional supplementary materials:  crystallographic information; 3D view; checkCIF report

## Figures and Tables

**Table 1:** Selected bond lengths (Å)

Cd1—O1	2.6353 (13)
Cd1—O2	2.2722 (13)
Cd1—O2^i^	2.5273 (12)
Cd1—O3	2.3739 (13)
Cd1—O4	2.3403 (13)
Cd1—O5	2.2987 (13)
Cd1—N1	2.3243 (15)

Symmetry code: (i) 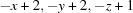.

**Table 2:** Hydrogen-bond geometry (Å, °)

*D*—H⋯*A*	*D*—H	H⋯*A*	*D*⋯*A*	*D*—H⋯*A*
N2—H2*A*⋯O1^ii^	0.86	2.10	2.931 (2)	162
N2—H2*B*⋯O4^i^	0.86	2.14	2.963 (2)	161
O5—H5*A*⋯O3^iii^	0.87	1.89	2.761 (2)	177
O5—H5*B*⋯O6^iv^	0.84	1.87	2.689 (2)	165
C3*A*—H3*A*⋯O3^i^	0.93	2.39	3.303 (3)	169
C11—H11⋯O5^iii^	0.93	2.60	3.481 (2)	159
C17—H17⋯O4^i^	0.93	2.45	3.286 (2)	150
C21—H21⋯O3^iii^	0.93	2.51	3.352 (3)	150

Symmetry codes: (i) 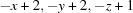; (ii) 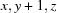; (iii) 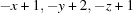; (iv) 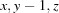.

**Fig. 1.**
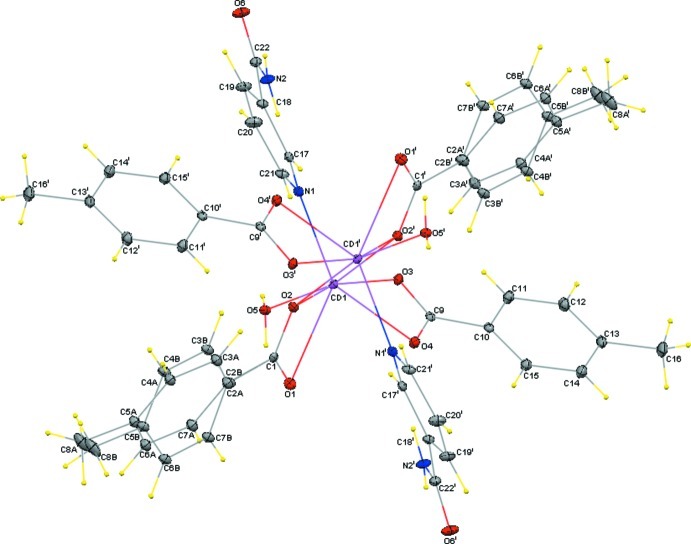
The molecular structure of the title compound with the atom-numbering scheme. Displacement ellipsoids are drawn at the 40% probability level. Primed atoms are generated by the symmetry operators: (') 2 - x, 2 - y, 1 - z.

## supplementary crystallographic information

### Comment

As a part of our ongoing investigation on transition metal complexes of nicotinamide (NA), one form of niacin (Krishnamachari, 1974), and/or the nicotinic acid derivative *N*,*N*-diethylnicotinamide (DENA), an important respiratory stimulant (Bigoli *et al.*, 1972), the title compound was synthesized and its crystal structure is reported herein.

The title compound, (I), consists of dimeric units located around a crystallographic symmetry centre and made up of two Cd cations, four 4-methylbenzoate (PMB) anions, two nicotinamide (NA) ligands and two water molecules (Fig. 1). Each Cd(II) unit is chelated by the carboxylate O atoms of the two PMB anions, and the two monomeric units are bridged through the two oxygen atoms of the two carboxylate groups about an inversion center. The coordination number of each Cd^II^ atom is seven. The Cd1···Cd1^i^ distance is 3.7796 (2) Å and O1-Cd1-O1^i^ angle is 76.19 (4)° (symmetry code: (i) -x, -y, 1 - z).

The average Cd-O bond length (Table 1) is 2.4080 (13) Å, and the Cd atom is displaced out of the least-squares planes of the carboxylate groups (O1/C1/O2) and (O3/C9/O4) by -0.5904 (1) and 0.3109 (1) Å, respectively. In (I), the O1-Cd1-O2 and O3-Cd1-O4 angles are 52.85 (4) and 55.62 (4) °, respectively. The corresponding O-M-O (where M is a metal) angles are 53.71 (4)° and 54.59 (4)° in [Cd_2_(HB)_4_(INA)_2_].4H_2_O (Zaman *et al.*, 2012), 55.71 (5)° and 117.52 (4)° in [Cd_2_(MAB)_4_(NA)_2_(H_2_O)_2_] (Hökelek *et al.*, 2010*b*), 55.96 (4)° and 53.78 (4)° in [Cd_2_(DMAB)_4_(NA)_2_(H_2_O)_2_] (Hökelek *et al.*, 2010*a*), 52.91 (4)° and 53.96 (4)° in [Cd(FB)_2_(INA)_2_(H_2_O)].H_2_O (Hökelek *et al.*, 2009*a*), 60.70 (4)° in [Co(DMAB)_2_(INA)(H_2_O)_2_] (Hökelek *et al.*, 2009*b*), 58.45 (9)° in [Mn(DMAB)_2_(INA)(H_2_O)_2_] (Hökelek *et al.*, 2009*c*), 60.03 (6)° in [Zn(MAB)_2_(INA)_2_].H_2_O (Hökelek *et al.*, 2009*d*), 58.3 (3)° in [Zn_2_(DENA)_2_(HB)_4_].2H_2_O (Hökelek & Necefoğlu, 1996) [where NA, INA, DENA, HB, FB, MAB and DMAB are nicotinamide, isonicotinamide, *N*,*N*-diethylnicotinamide, 3- or 4-hydroxybenzoate, 4-formylbenzoate, 4-methylaminobenzoate and 4-dimethylaminobenzoate, respectively] and 55.2 (1)° in [Cu(Asp)_2_(py)_2_] (where Asp is acetylsalicylate and py is pyridine) (Greenaway *et al.*, 1984).

The dihedral angle between the planar carboxylate group (O3/C9/O4) and the adjacent benzene ring B (C10-C15) is 7.21 (19) °, while those between rings B, C (N1/C17-C21), D (Cd1/O1/O2/C1) and E (Cd1/O3/O4/C9) are B/C = 59.55 (7) and D/E = 63.01 (5)°.

In the crystal structure, intermolecular O-H···O, N-H···O and C-H···O hydrogen bonds (Table 2) link the molecules into a three-dimensional network, in which they may be effective in the stabilization of the structure.

### Experimental

The title compound was prepared by the reaction of 3CdSO_4_.8H_2_O (1.285 g, 5 mmol) in H_2_O (50 ml) and NA (1.220 g, 10 mmol) in H_2_O (20 ml) with sodium 4-methylbenzoate (1.580 g, 10 mmol) in H_2_O (400 ml). The mixture was filtered and set aside to crystallize at ambient temperature for three weeks, giving colorless single crystals.

### Refinement

Atoms H51 and H52 (for H_2_O) were located in a difference Fourier map and their positions were kept fixed during the refinement process. The remaining H atoms were positioned geometrically with N—H = 0.86 Å (for NH_2_), C—H = 0.93 and 0.96 Å for aromatic and methyl H atoms and constrained to ride on their parent atoms, with *U*_iso_(H) = *xU*_eq_(C,N,O), where *x* = 1.5 for methyl and water H atoms and *x* = 1.2 for NH_2_ and aromatic H atoms. In the benzene ring A (C2-C7), the C2, C3, C4, C5 C6, C7 and the attached H3, H4, H6, H7 atoms, respectively, together with the C8, H81, H82 and H83 atoms of the methyl group attached at C5 are disordered over two orientations. During the refinement process the disordered C2A, C3A, H3A, C4A, H4A, C5A, C6A, H6A, C7A, H7A, C8A, H8A1, H8A2, H8A3 and C2B, C3B, H3B, C4B, H4B, C5B, C6B, H6B, C7B, H7B, C8B, H8B1, H8B2, H8B3 atoms were refined with occupancies ratios of 0.50:0.50.

### Crystal data

**Table:**

[Cd_2_(C_8_H_7_O_2_)_4_(C_6_H_6_N_2_O)_2_(H_2_O)_2_]	*Z* = 1
*M**_r_* = 1045.65	*F*(000) = 528
Triclinic, *P*1	*D*_x_ = 1.637 Mg m^−^^3^
Hall symbol: -P 1	Mo *K*α radiation, λ = 0.71073 Å
*a* = 9.5935 (2) Å	Cell parameters from 9926 reflections
*b* = 10.3084 (2) Å	θ = 2.3–28.5°
*c* = 12.6606 (3) Å	µ = 1.07 mm^−^^1^
α = 68.326 (3)°	*T* = 100 K
β = 74.999 (3)°	Block, colorless
γ = 66.916 (2)°	0.36 × 0.31 × 0.28 mm
*V* = 1060.81 (5) Å^3^	

### Data collection

**Table:**

Bruker Kappa APEXII CCD area-detector diffractometer	4169 independent reflections
Radiation source: fine-focus sealed tube	4085 reflections with *I* > 2σ(*I*)
Graphite monochromator	*R*_int_ = 0.021
φ and ω scans	θ_max_ = 26.0°, θ_min_ = 1.8°
Absorption correction: multi-scan (*SADABS*; Bruker, 2005)	*h* = −11→11
*T*_min_ = 0.687, *T*_max_ = 0.741	*k* = −12→12
16908 measured reflections	*l* = −15→15

### Refinement

**Table:**

Refinement on *F*^2^	Primary atom site location: structure-invariant direct methods
Least-squares matrix: full	Secondary atom site location: difference Fourier map
*R*[*F*^2^ > 2σ(*F*^2^)] = 0.019	Hydrogen site location: inferred from neighbouring sites
*wR*(*F*^2^) = 0.048	H-atom parameters constrained
*S* = 1.05	*w* = 1/[σ^2^(*F*_o_^2^) + (0.0193*P*)^2^ + 1.1679*P*] where *P* = (*F*_o_^2^ + 2*F*_c_^2^)/3
4169 reflections	(Δ/σ)_max_ = 0.002
256 parameters	Δρ_max_ = 1.08 e Å^−^^3^
0 restraints	Δρ_min_ = −0.72 e Å^−^^3^

### Special details

**Table:**

Geometry. All esds (except the esd in the dihedral angle between two l.s. planes) are estimated using the full covariance matrix. The cell esds are taken into account individually in the estimation of esds in distances, angles and torsion angles; correlations between esds in cell parameters are only used when they are defined by crystal symmetry. An approximate (isotropic) treatment of cell esds is used for estimating esds involving l.s. planes.
Refinement. Refinement of F^2^ against ALL reflections. The weighted R-factor wR and goodness of fit S are based on F^2^, conventional R-factors R are based on F, with F set to zero for negative F^2^. The threshold expression of F^2^ > 2sigma(F^2^) is used only for calculating R-factors(gt) etc. and is not relevant to the choice of reflections for refinement. R-factors based on F^2^ are statistically about twice as large as those based on F, and R- factors based on ALL data will be even larger.

### Fractional atomic coordinates and isotropic or equivalent isotropic displacement parameters (Å^2^)

**Table:**

	*x*	*y*	*z*	*U*_iso_*/*U*_eq_	Occ. (<1)
Cd1	0.817156 (14)	0.963852 (13)	0.512013 (10)	0.01282 (5)	
O1	0.96132 (15)	0.68775 (14)	0.62253 (11)	0.0203 (3)	
O2	1.01690 (14)	0.88132 (13)	0.61152 (10)	0.0160 (3)	
O3	0.66922 (14)	1.01399 (14)	0.36876 (11)	0.0172 (3)	
O4	0.90596 (15)	0.86161 (14)	0.36017 (11)	0.0181 (3)	
O5	0.64150 (14)	0.86318 (13)	0.64128 (11)	0.0169 (3)	
H5A	0.5440	0.8987	0.6376	0.025*	
H5B	0.6577	0.7756	0.6444	0.025*	
O6	0.63943 (16)	1.59642 (15)	0.65749 (14)	0.0291 (3)	
N1	0.67632 (17)	1.17891 (16)	0.56260 (13)	0.0151 (3)	
N2	0.87749 (18)	1.44037 (18)	0.62850 (15)	0.0220 (3)	
H2A	0.9187	1.4975	0.6345	0.026*	
H2B	0.9342	1.3578	0.6157	0.026*	
C1	1.02645 (19)	0.74445 (19)	0.65768 (15)	0.0140 (3)	
C2A	1.1134 (3)	0.6536 (3)	0.7611 (2)	0.0220 (5)	0.50
C3A	1.2108 (4)	0.7037 (3)	0.7882 (3)	0.0220 (5)	0.50
H3A	1.2330	0.7878	0.7384	0.026*	0.50
C4A	1.2749 (3)	0.6283 (4)	0.8898 (3)	0.0220 (5)	0.50
H4A	1.3400	0.6618	0.9079	0.026*	0.50
C5A	1.2417 (3)	0.5027 (3)	0.9642 (2)	0.0220 (5)	0.50
C6A	1.1444 (4)	0.4525 (3)	0.9370 (2)	0.0220 (5)	0.50
H6A	1.1222	0.3685	0.9868	0.026*	0.50
C7A	1.0803 (3)	0.5280 (3)	0.8355 (2)	0.0220 (5)	0.50
H7A	1.0151	0.4945	0.8173	0.026*	0.50
C8A	1.305 (3)	0.422 (4)	1.079 (3)	0.042 (3)	0.50
H8A1	1.3720	0.4670	1.0844	0.064*	0.50
H8A2	1.3608	0.3199	1.0830	0.064*	0.50
H8A3	1.2221	0.4272	1.1403	0.064*	0.50
C2B	1.1134 (3)	0.6556 (3)	0.7575 (2)	0.0196 (5)	0.50
C3B	1.1715 (4)	0.7249 (2)	0.8029 (3)	0.0196 (5)	0.50
H3B	1.1594	0.8249	0.7691	0.024*	0.50
C4B	1.2478 (3)	0.6447 (3)	0.8986 (3)	0.0196 (5)	0.50
H4B	1.2867	0.6911	0.9289	0.024*	0.50
C5B	1.2659 (3)	0.4953 (3)	0.9490 (2)	0.0196 (5)	0.50
C6B	1.2078 (4)	0.4260 (2)	0.9036 (2)	0.0196 (5)	0.50
H6B	1.2199	0.3260	0.9373	0.024*	0.50
C7B	1.1315 (3)	0.5061 (3)	0.8079 (2)	0.0196 (5)	0.50
H7B	1.0927	0.4597	0.7776	0.024*	0.50
C8B	1.340 (3)	0.414 (4)	1.059 (3)	0.042 (3)	0.50
H8B1	1.2836	0.3522	1.1132	0.064*	0.50
H8B2	1.3403	0.4842	1.0919	0.064*	0.50
H8B3	1.4436	0.3532	1.0422	0.064*	0.50
C9	0.7838 (2)	0.93699 (19)	0.31593 (15)	0.0147 (3)	
C10	0.7771 (2)	0.9423 (2)	0.19762 (15)	0.0167 (4)	
C11	0.6466 (2)	1.0303 (2)	0.14489 (17)	0.0236 (4)	
H11	0.5596	1.0809	0.1854	0.028*	
C12	0.6455 (3)	1.0431 (2)	0.03214 (18)	0.0266 (4)	
H12	0.5574	1.1024	−0.0022	0.032*	
C13	0.7736 (3)	0.9690 (2)	−0.03055 (16)	0.0244 (4)	
C14	0.9032 (3)	0.8808 (2)	0.02313 (18)	0.0269 (4)	
H14	0.9898	0.8298	−0.0172	0.032*	
C15	0.9059 (2)	0.8675 (2)	0.13545 (17)	0.0222 (4)	
H15	0.9941	0.8083	0.1696	0.027*	
C16	0.7713 (3)	0.9837 (3)	−0.15339 (18)	0.0347 (5)	
H16A	0.7736	0.8918	−0.1577	0.052*	
H16B	0.8589	1.0080	−0.2006	0.052*	
H16C	0.6798	1.0605	−0.1799	0.052*	
C17	0.7412 (2)	1.25977 (19)	0.58365 (15)	0.0149 (3)	
H17	0.8471	1.2344	0.5697	0.018*	
C18	0.6572 (2)	1.37934 (19)	0.62518 (16)	0.0168 (4)	
C19	0.4990 (2)	1.4139 (2)	0.64957 (19)	0.0250 (4)	
H19	0.4395	1.4915	0.6796	0.030*	
C20	0.4319 (2)	1.3308 (2)	0.6283 (2)	0.0277 (5)	
H20	0.3265	1.3514	0.6443	0.033*	
C21	0.5235 (2)	1.2166 (2)	0.58314 (17)	0.0203 (4)	
H21	0.4772	1.1637	0.5663	0.024*	
C22	0.7261 (2)	1.4797 (2)	0.63911 (16)	0.0186 (4)	

### Atomic displacement parameters (Å^2^)

**Table:**

	*U*^11^	*U*^22^	*U*^33^	*U*^12^	*U*^13^	*U*^23^
Cd1	0.01161 (7)	0.01484 (7)	0.01396 (7)	−0.00428 (5)	−0.00216 (5)	−0.00624 (5)
O1	0.0207 (7)	0.0209 (7)	0.0224 (7)	−0.0098 (5)	−0.0069 (5)	−0.0040 (5)
O2	0.0167 (6)	0.0148 (6)	0.0150 (6)	−0.0054 (5)	−0.0026 (5)	−0.0022 (5)
O3	0.0158 (6)	0.0205 (6)	0.0163 (6)	−0.0058 (5)	−0.0022 (5)	−0.0070 (5)
O4	0.0169 (6)	0.0194 (6)	0.0190 (6)	−0.0036 (5)	−0.0053 (5)	−0.0075 (5)
O5	0.0152 (6)	0.0131 (6)	0.0217 (7)	−0.0044 (5)	−0.0018 (5)	−0.0052 (5)
O6	0.0198 (7)	0.0197 (7)	0.0533 (10)	−0.0052 (6)	−0.0021 (7)	−0.0203 (7)
N1	0.0155 (7)	0.0142 (7)	0.0155 (7)	−0.0054 (6)	−0.0033 (6)	−0.0030 (6)
N2	0.0172 (8)	0.0180 (8)	0.0365 (10)	−0.0057 (6)	−0.0044 (7)	−0.0142 (7)
C1	0.0095 (8)	0.0157 (8)	0.0147 (8)	−0.0035 (7)	0.0021 (6)	−0.0054 (7)
C2A	0.0206 (9)	0.0223 (10)	0.0217 (10)	−0.0072 (7)	−0.0045 (7)	−0.0038 (8)
C3A	0.0206 (9)	0.0223 (10)	0.0217 (10)	−0.0072 (7)	−0.0045 (7)	−0.0038 (8)
C4A	0.0206 (9)	0.0223 (10)	0.0217 (10)	−0.0072 (7)	−0.0045 (7)	−0.0038 (8)
C5A	0.0206 (9)	0.0223 (10)	0.0217 (10)	−0.0072 (7)	−0.0045 (7)	−0.0038 (8)
C6A	0.0206 (9)	0.0223 (10)	0.0217 (10)	−0.0072 (7)	−0.0045 (7)	−0.0038 (8)
C7A	0.0206 (9)	0.0223 (10)	0.0217 (10)	−0.0072 (7)	−0.0045 (7)	−0.0038 (8)
C8A	0.045 (9)	0.042 (4)	0.036 (8)	−0.020 (7)	−0.026 (6)	0.012 (5)
C2B	0.0151 (8)	0.0173 (10)	0.0238 (10)	−0.0035 (6)	−0.0062 (7)	−0.0027 (8)
C3B	0.0151 (8)	0.0173 (10)	0.0238 (10)	−0.0035 (6)	−0.0062 (7)	−0.0027 (8)
C4B	0.0151 (8)	0.0173 (10)	0.0238 (10)	−0.0035 (6)	−0.0062 (7)	−0.0027 (8)
C5B	0.0151 (8)	0.0173 (10)	0.0238 (10)	−0.0035 (6)	−0.0062 (7)	−0.0027 (8)
C6B	0.0151 (8)	0.0173 (10)	0.0238 (10)	−0.0035 (6)	−0.0062 (7)	−0.0027 (8)
C7B	0.0151 (8)	0.0173 (10)	0.0238 (10)	−0.0035 (6)	−0.0062 (7)	−0.0027 (8)
C8B	0.045 (9)	0.042 (4)	0.036 (8)	−0.020 (7)	−0.026 (6)	0.012 (5)
C9	0.0188 (9)	0.0142 (8)	0.0145 (8)	−0.0106 (7)	−0.0021 (7)	−0.0023 (7)
C10	0.0223 (9)	0.0166 (8)	0.0148 (9)	−0.0112 (7)	−0.0028 (7)	−0.0033 (7)
C11	0.0226 (10)	0.0288 (10)	0.0200 (10)	−0.0069 (8)	−0.0040 (8)	−0.0090 (8)
C12	0.0306 (11)	0.0297 (11)	0.0206 (10)	−0.0097 (9)	−0.0094 (8)	−0.0051 (8)
C13	0.0385 (12)	0.0239 (10)	0.0152 (9)	−0.0165 (9)	−0.0040 (8)	−0.0039 (8)
C14	0.0320 (11)	0.0288 (10)	0.0208 (10)	−0.0107 (9)	0.0012 (8)	−0.0109 (8)
C15	0.0243 (10)	0.0229 (9)	0.0201 (10)	−0.0084 (8)	−0.0034 (8)	−0.0063 (8)
C16	0.0517 (15)	0.0386 (12)	0.0174 (10)	−0.0179 (11)	−0.0054 (10)	−0.0086 (9)
C17	0.0149 (9)	0.0154 (8)	0.0143 (8)	−0.0061 (7)	−0.0021 (7)	−0.0029 (7)
C18	0.0159 (9)	0.0137 (8)	0.0208 (9)	−0.0059 (7)	−0.0024 (7)	−0.0042 (7)
C19	0.0169 (10)	0.0172 (9)	0.0415 (12)	−0.0037 (8)	−0.0008 (9)	−0.0137 (9)
C20	0.0129 (9)	0.0203 (9)	0.0503 (14)	−0.0045 (8)	−0.0028 (9)	−0.0131 (9)
C21	0.0172 (9)	0.0156 (8)	0.0298 (10)	−0.0073 (7)	−0.0056 (8)	−0.0050 (8)
C22	0.0199 (9)	0.0153 (8)	0.0214 (9)	−0.0063 (7)	−0.0028 (7)	−0.0060 (7)

### Geometric parameters (Å, º)

**Table:**

Cd1—O1	2.6353 (13)	C2B—C7B	1.3900
Cd1—O2	2.2722 (13)	C3B—C4B	1.3900
Cd1—O2^i^	2.5273 (12)	C3B—H3B	0.9300
Cd1—O3	2.3739 (13)	C4B—C5B	1.3900
Cd1—O4	2.3403 (13)	C4B—H4B	0.9300
Cd1—O5	2.2987 (13)	C5B—C6B	1.3900
Cd1—N1	2.3243 (15)	C5B—C8B	1.53 (3)
Cd1—C9	2.7076 (17)	C6B—C7B	1.3900
N1—C17	1.345 (2)	C6B—H6B	0.9300
N1—C21	1.341 (2)	C7B—H7B	0.9300
N2—C22	1.330 (2)	C8B—H8B1	0.9600
N2—H2A	0.8600	C8B—H8B2	0.9600
N2—H2B	0.8600	C8B—H8B3	0.9600
O1—C1	1.246 (2)	C9—C10	1.496 (2)
O2—C1	1.288 (2)	C10—C11	1.390 (3)
O2—Cd1^i^	2.5273 (12)	C10—C15	1.392 (3)
O3—C9	1.270 (2)	C11—C12	1.387 (3)
O4—C9	1.263 (2)	C11—H11	0.9300
O5—H5A	0.8678	C12—C13	1.389 (3)
O5—H5B	0.8411	C12—H12	0.9300
O6—C22	1.231 (2)	C13—C14	1.389 (3)
C1—C2B	1.491 (3)	C13—C16	1.511 (3)
C1—C2A	1.528 (3)	C14—C15	1.383 (3)
C2A—C3A	1.3900	C14—H14	0.9300
C2A—C7A	1.3900	C15—H15	0.9300
C3A—C4A	1.3900	C16—H16A	0.9600
C3A—H3A	0.9300	C16—H16B	0.9600
C4A—C5A	1.3900	C16—H16C	0.9600
C4A—H4A	0.9300	C17—C18	1.388 (2)
C5A—C6A	1.3900	C17—H17	0.9300
C5A—C8A	1.53 (3)	C18—C19	1.393 (3)
C6A—C7A	1.3900	C18—C22	1.506 (2)
C6A—H6A	0.9300	C19—C20	1.383 (3)
C7A—H7A	0.9300	C19—H19	0.9300
C8A—H8A1	0.9600	C20—C21	1.382 (3)
C8A—H8A2	0.9600	C20—H20	0.9300
C8A—H8A3	0.9600	C21—H21	0.9300
C2B—C3B	1.3900		
			
O1—Cd1—C9	101.80 (5)	C4B—C3B—C2B	120.0
O2—Cd1—O1	52.85 (4)	C4B—C3B—H3B	120.0
O2^i^—Cd1—O1	116.34 (4)	C2B—C3B—H3B	120.0
O2—Cd1—O2^i^	76.22 (5)	C5B—C4B—C3B	120.0
O2—Cd1—O3	162.16 (4)	C5B—C4B—H4B	120.0
O2—Cd1—O4	106.73 (4)	C3B—C4B—H4B	120.0
O2—Cd1—O5	103.70 (4)	C4B—C5B—C6B	120.0
O2—Cd1—N1	99.20 (5)	C4B—C5B—C8B	118.4 (14)
O2—Cd1—C9	134.23 (5)	C6B—C5B—C8B	121.5 (14)
O2^i^—Cd1—C9	86.97 (5)	C7B—C6B—C5B	120.0
O3—Cd1—O1	119.67 (4)	C7B—C6B—H6B	120.0
O3—Cd1—O2^i^	96.74 (4)	C5B—C6B—H6B	120.0
O3—Cd1—C9	27.97 (5)	C6B—C7B—C2B	120.0
O4—Cd1—O1	80.08 (4)	C6B—C7B—H7B	120.0
O4—Cd1—O2^i^	81.30 (4)	C2B—C7B—H7B	120.0
O4—Cd1—O3	55.62 (4)	C5B—C8B—H8B1	109.5
O4—Cd1—C9	27.78 (5)	C5B—C8B—H8B2	109.5
O5—Cd1—O1	71.23 (4)	H8B1—C8B—H8B2	109.5
O5—Cd1—O2^i^	167.88 (4)	C5B—C8B—H8B3	109.5
O5—Cd1—O3	86.63 (4)	H8B1—C8B—H8B3	109.5
O5—Cd1—O4	109.99 (5)	H8B2—C8B—H8B3	109.5
O5—Cd1—N1	86.22 (5)	O4—C9—O3	120.53 (16)
O5—Cd1—C9	101.04 (5)	O4—C9—C10	119.65 (16)
N1—Cd1—O1	135.38 (5)	O3—C9—C10	119.74 (16)
N1—Cd1—O2^i^	81.86 (5)	O4—C9—Cd1	59.71 (9)
N1—Cd1—O3	95.92 (5)	O3—C9—Cd1	61.25 (9)
N1—Cd1—O4	144.53 (5)	C10—C9—Cd1	170.35 (12)
N1—Cd1—C9	120.41 (5)	C11—C10—C15	118.92 (17)
C1—O1—Cd1	84.10 (10)	C11—C10—C9	120.69 (17)
Cd1—O2—Cd1^i^	103.78 (5)	C15—C10—C9	120.21 (17)
C1—O2—Cd1	99.86 (11)	C12—C11—C10	120.23 (19)
C1—O2—Cd1^i^	139.66 (11)	C12—C11—H11	119.9
C9—O3—Cd1	90.78 (10)	C10—C11—H11	119.9
C9—O4—Cd1	92.51 (10)	C11—C12—C13	121.2 (2)
Cd1—O5—H5A	124.2	C11—C12—H12	119.4
Cd1—O5—H5B	113.2	C13—C12—H12	119.4
H5A—O5—H5B	99.0	C14—C13—C12	118.05 (18)
C17—N1—Cd1	123.00 (12)	C14—C13—C16	121.1 (2)
C21—N1—Cd1	118.75 (12)	C12—C13—C16	120.8 (2)
C21—N1—C17	117.90 (16)	C15—C14—C13	121.3 (2)
C22—N2—H2A	120.0	C15—C14—H14	119.4
C22—N2—H2B	120.0	C13—C14—H14	119.4
H2A—N2—H2B	120.0	C14—C15—C10	120.27 (19)
O1—C1—O2	121.27 (16)	C14—C15—H15	119.9
O1—C1—C2B	120.30 (18)	C10—C15—H15	119.9
O2—C1—C2B	118.40 (18)	C13—C16—H16A	109.5
O1—C1—C2A	120.12 (19)	C13—C16—H16B	109.5
O2—C1—C2A	118.55 (18)	H16A—C16—H16B	109.5
C3A—C2A—C7A	120.0	C13—C16—H16C	109.5
C3A—C2A—C1	121.0 (2)	H16A—C16—H16C	109.5
C7A—C2A—C1	118.6 (2)	H16B—C16—H16C	109.5
C4A—C3A—C2A	120.0	N1—C17—C18	122.98 (17)
C4A—C3A—H3A	120.0	N1—C17—H17	118.5
C2A—C3A—H3A	120.0	C18—C17—H17	118.5
C3A—C4A—C5A	120.0	C17—C18—C19	118.31 (17)
C3A—C4A—H4A	120.0	C17—C18—C22	123.76 (16)
C5A—C4A—H4A	120.0	C19—C18—C22	117.83 (16)
C6A—C5A—C4A	120.0	C20—C19—C18	118.87 (18)
C6A—C5A—C8A	118.9 (13)	C20—C19—H19	120.6
C4A—C5A—C8A	121.0 (13)	C18—C19—H19	120.6
C7A—C6A—C5A	120.0	C21—C20—C19	119.12 (18)
C7A—C6A—H6A	120.0	C21—C20—H20	120.4
C5A—C6A—H6A	120.0	C19—C20—H20	120.4
C6A—C7A—C2A	120.0	N1—C21—C20	122.76 (17)
C6A—C7A—H7A	120.0	N1—C21—H21	118.6
C2A—C7A—H7A	120.0	C20—C21—H21	118.6
C3B—C2B—C7B	120.0	O6—C22—N2	122.79 (17)
C3B—C2B—C1	119.3 (2)	O6—C22—C18	118.32 (17)
C7B—C2B—C1	120.6 (2)	N2—C22—C18	118.87 (16)
			
O2—Cd1—O1—C1	7.87 (10)	Cd1—O2—C1—C2B	−162.66 (17)
O2^i^—Cd1—O1—C1	52.76 (11)	Cd1^i^—O2—C1—C2B	72.0 (2)
O3—Cd1—O1—C1	168.40 (10)	Cd1—O3—C9—O4	7.53 (16)
O4—Cd1—O1—C1	127.81 (10)	Cd1—O3—C9—C10	−168.99 (14)
O5—Cd1—O1—C1	−116.97 (11)	Cd1—O4—C9—O3	−7.64 (16)
N1—Cd1—O1—C1	−53.31 (12)	Cd1—O4—C9—C10	168.88 (13)
C9—Cd1—O1—C1	145.24 (10)	Cd1—N1—C17—C18	−173.17 (13)
O1—Cd1—O2—Cd1^i^	139.37 (7)	C21—N1—C17—C18	−0.1 (3)
O1—Cd1—O2—C1	−7.69 (9)	Cd1—N1—C21—C20	171.10 (16)
O2^i^—Cd1—O2—Cd1^i^	0.0	C17—N1—C21—C20	−2.3 (3)
O2^i^—Cd1—O2—C1	−147.05 (12)	O1—C1—C2A—C3A	167.42 (17)
O3—Cd1—O2—Cd1^i^	68.42 (15)	O1—C1—C2A—C7A	−19.9 (3)
O3—Cd1—O2—C1	−78.64 (18)	O2—C1—C2A—C3A	−15.5 (3)
O4—Cd1—O2—Cd1^i^	76.33 (5)	O2—C1—C2A—C7A	157.16 (18)
O4—Cd1—O2—C1	−70.73 (11)	C2B—C1—C2A—C3A	64 (16)
O5—Cd1—O2—Cd1^i^	−167.52 (4)	C2B—C1—C2A—C7A	−124 (16)
O5—Cd1—O2—C1	45.42 (11)	C1—C2A—C3A—C4A	172.5 (3)
N1—Cd1—O2—Cd1^i^	−79.20 (5)	C7A—C2A—C3A—C4A	0.0
N1—Cd1—O2—C1	133.75 (10)	C1—C2A—C7A—C6A	−172.7 (3)
C9—Cd1—O2—Cd1^i^	71.66 (7)	C3A—C2A—C7A—C6A	0.0
C9—Cd1—O2—C1	−75.39 (12)	C2A—C3A—C4A—C5A	0.0
O1—Cd1—O3—C9	−55.17 (11)	C3A—C4A—C5A—C6A	0.0
O2—Cd1—O3—C9	5.0 (2)	C3A—C4A—C5A—C8A	−177.3 (11)
O2^i^—Cd1—O3—C9	70.38 (10)	C4A—C5A—C6A—C7A	0.0
O4—Cd1—O3—C9	−4.23 (9)	C8A—C5A—C6A—C7A	177.4 (11)
O5—Cd1—O3—C9	−121.31 (10)	C5A—C6A—C7A—C2A	0.0
N1—Cd1—O3—C9	152.85 (10)	O1—C1—C2B—C3B	−173.29 (17)
O1—Cd1—O4—C9	141.03 (10)	O1—C1—C2B—C7B	4.2 (3)
O2—Cd1—O4—C9	−172.81 (10)	O2—C1—C2B—C3B	4.7 (3)
O2^i^—Cd1—O4—C9	−100.13 (10)	O2—C1—C2B—C7B	−177.77 (17)
O3—Cd1—O4—C9	4.26 (9)	C2A—C1—C2B—C3B	−97 (16)
O5—Cd1—O4—C9	75.32 (10)	C2A—C1—C2B—C7B	81 (16)
N1—Cd1—O4—C9	−37.61 (14)	C1—C2B—C3B—C4B	177.6 (2)
O1—Cd1—N1—C17	77.16 (15)	C7B—C2B—C3B—C4B	0.0
O1—Cd1—N1—C21	−95.89 (14)	C1—C2B—C7B—C6B	−177.5 (3)
O2—Cd1—N1—C17	32.13 (14)	C3B—C2B—C7B—C6B	0.0
O2^i^—Cd1—N1—C17	−42.39 (13)	C2B—C3B—C4B—C5B	0.0
O2—Cd1—N1—C21	−140.92 (13)	C3B—C4B—C5B—C6B	0.0
O2^i^—Cd1—N1—C21	144.57 (14)	C3B—C4B—C5B—C8B	−175.6 (9)
O3—Cd1—N1—C17	−138.37 (13)	C4B—C5B—C6B—C7B	0.0
O3—Cd1—N1—C21	48.58 (14)	C8B—C5B—C6B—C7B	175.5 (9)
O4—Cd1—N1—C17	−104.74 (14)	C5B—C6B—C7B—C2B	0.0
O4—Cd1—N1—C21	82.21 (16)	O3—C9—C10—C11	−0.2 (3)
O5—Cd1—N1—C17	135.41 (14)	O3—C9—C10—C15	174.98 (16)
O5—Cd1—N1—C21	−37.63 (14)	O4—C9—C10—C11	−176.73 (17)
C9—Cd1—N1—C17	−124.00 (13)	O4—C9—C10—C15	−1.6 (3)
C9—Cd1—N1—C21	62.95 (15)	C9—C10—C11—C12	175.15 (18)
O1—Cd1—C9—O3	133.23 (9)	C15—C10—C11—C12	−0.1 (3)
O1—Cd1—C9—O4	−39.26 (10)	C9—C10—C15—C14	−175.38 (17)
O2—Cd1—C9—O3	−177.88 (8)	C11—C10—C15—C14	−0.1 (3)
O2^i^—Cd1—C9—O3	−110.49 (10)	C10—C11—C12—C13	0.0 (3)
O2—Cd1—C9—O4	9.63 (13)	C11—C12—C13—C14	0.2 (3)
O2^i^—Cd1—C9—O4	77.02 (10)	C11—C12—C13—C16	180.00 (19)
O3—Cd1—C9—O4	−172.49 (16)	C12—C13—C14—C15	−0.4 (3)
O4—Cd1—C9—O3	172.49 (16)	C16—C13—C14—C15	179.8 (2)
O5—Cd1—C9—O3	60.34 (10)	C13—C14—C15—C10	0.4 (3)
O5—Cd1—C9—O4	−112.15 (10)	N1—C17—C18—C19	2.1 (3)
N1—Cd1—C9—O3	−31.76 (11)	N1—C17—C18—C22	−174.19 (16)
N1—Cd1—C9—O4	155.75 (9)	C17—C18—C19—C20	−1.9 (3)
Cd1—O1—C1—O2	−13.02 (15)	C22—C18—C19—C20	174.68 (19)
Cd1—O1—C1—C2A	164.00 (18)	C17—C18—C22—O6	167.60 (18)
Cd1—O1—C1—C2B	164.90 (18)	C17—C18—C22—N2	−10.9 (3)
Cd1—O2—C1—O1	15.30 (18)	C19—C18—C22—O6	−8.8 (3)
Cd1^i^—O2—C1—O1	−110.02 (19)	C19—C18—C22—N2	172.76 (19)
Cd1—O2—C1—C2A	−161.77 (16)	C18—C19—C20—C21	−0.3 (3)
Cd1^i^—O2—C1—C2A	72.9 (2)	C19—C20—C21—N1	2.5 (3)

Symmetry code: (i) −*x*+2, −*y*+2, −*z*+1.

### Hydrogen-bond geometry (Å, º)

**Table:**

*D*—H···*A*	*D*—H	H···*A*	*D*···*A*	*D*—H···*A*
N2—H2*A*···O1^ii^	0.86	2.10	2.931 (2)	162
N2—H2*B*···O4^i^	0.86	2.14	2.963 (2)	161
O5—H5*A*···O3^iii^	0.87	1.89	2.761 (2)	177
O5—H5*B*···O6^iv^	0.84	1.87	2.689 (2)	165
C3*A*—H3*A*···O3^i^	0.93	2.39	3.303 (3)	169
C11—H11···O5^iii^	0.93	2.60	3.481 (2)	159
C17—H17···O4^i^	0.93	2.45	3.286 (2)	150
C21—H21···O3^iii^	0.93	2.51	3.352 (3)	150

Symmetry codes: (i) −*x*+2, −*y*+2, −*z*+1; (ii) *x*, *y*+1, *z*; (iii) −*x*+1, −*y*+2, −*z*+1; (iv) *x*, *y*−1, *z*.
